# Supplementary material for: Improving the sensitivity of in vivo CRISPR off-target detection with DISCOVER-Seq+
Source: Nat Methods. 2023 Apr 6;20(5):706–13. doi: 10.1038/s41592-023-01840-z (PMC10172116; doi:10.1038/s41592-023-01840-z)
Supplement: Supplementary file 1 — Reporting Summary [file 41592_2023_1840_MOESM1_ESM.pdf]

nature research

Corresponding author(s): Taekjip Ha

Last updated by author(s): Jan 8, 2023

Reporting Summary

Nature Research wishes to improve the reproducibility of the work that we publish. This form provides structure for consistency and transparency in reporting. For further information on Nature Research policies, see our Editorial Policies and the Editorial Policy Checklist.

Statistics

For all statistical analyses, confirm that the following items are present in the figure legend, table legend, main text, or Methods section.

n/a	Confirmed	
☐	☒	The exact sample size (*n*) for each experimental group/condition, given as a discrete number and unit of measurement
☐	☒	A statement on whether measurements were taken from distinct samples or whether the same sample was measured repeatedly
☐	☒	The statistical test(s) used AND whether they are one- or two-sided<br>*Only common tests should be described solely by name; describe more complex techniques in the Methods section.*
☐	☒	A description of all covariates tested
☐	☒	A description of any assumptions or corrections, such as tests of normality and adjustment for multiple comparisons
☐	☒	A full description of the statistical parameters including central tendency (e.g. means) or other basic estimates (e.g. regression coefficient) AND variation (e.g. standard deviation) or associated estimates of uncertainty (e.g. confidence intervals)
☐	☒	For null hypothesis testing, the test statistic (e.g. *F*, *t*, *r*) with confidence intervals, effect sizes, degrees of freedom and *P* value noted<br>*Give P values as exact values whenever suitable.*
☒	☐	For Bayesian analysis, information on the choice of priors and Markov chain Monte Carlo settings
☒	☐	For hierarchical and complex designs, identification of the appropriate level for tests and full reporting of outcomes
☐	☒	Estimates of effect sizes (e.g. Cohen's *d*, Pearson's *r*), indicating how they were calculated

Our web collection on statistics for biologists contains articles on many of the points above.

Software and code

Policy information about availability of computer code

Data collection	No code was used for collecting the data used in this study.
Data analysis	FlowJo v.10, bcl2fastq2, bowtie2, macs2, samtools1.10, scikit-learn1.1, custom code for data analysis (https://github.com/rogerzou/DseqPlus), Blender (version 9cb8a72; Dec 12, 2019) for generating list of off-target sites from sequencing data (https://github.com/staciawyman/blender)

For manuscripts utilizing custom algorithms or software that are central to the research but not yet described in published literature, software must be made available to editors and reviewers. We strongly encourage code deposition in a community repository (e.g. GitHub). See the Nature Research guidelines for submitting code & software for further information.

Data

Policy information about availability of data

All manuscripts must include a data availability statement. This statement should provide the following information, where applicable:

- Accession codes, unique identifiers, or web links for publicly available datasets
- A list of figures that have associated raw data
- A description of any restrictions on data availability

Deep-sequencing data that support the findings of this study have been deposited in Sequence Read Archive under BioProject accession PRJNA801688. Sequencing data was analyzed using the hg38 genome assembly (https://www.ncbi.nlm.nih.gov/assembly/GCF_000001405.26). Source data have been provided in Source Data.

Field-specific reporting

Please select the one below that is the best fit for your research. If you are not sure, read the appropriate sections before making your selection.

☒ Life sciences ☐ Behavioural & social sciences ☐ Ecological, evolutionary & environmental sciences

For a reference copy of the document with all sections, see nature.com/documents/nr-reporting-summary-flat.pdf

Life sciences study design

All studies must disclose on these points even when the disclosure is negative.

Sample size	No statistical method was used to predetermine sample size.
Data exclusions	No data was excluded from the analysis.
Replication	All attempts at replication were successful (triplicates).
Randomization	Allocation was randomly selected.
Blinding	Not relevant because no group allocation was performed in this study.

Reporting for specific materials, systems and methods

We require information from authors about some types of materials, experimental systems and methods used in many studies. Here, indicate whether each material, system or method listed is relevant to your study. If you are not sure if a list item applies to your research, read the appropriate section before selecting a response.

Materials & experimental systems

n/a	Involved in the study	
☐	☒	Antibodies
☐	☒	Eukaryotic cell lines
☒	☐	Palaeontology and archaeology
☐	☒	Animals and other organisms
☒	☐	Human research participants
☒	☐	Clinical data
☒	☐	Dual use research of concern

Methods

n/a	Involved in the study	
☐	☒	ChIP-seq
☐	☒	Flow cytometry
☒	☐	MRI-based neuroimaging

Antibodies

Antibodies used	rabbit anti-MRE11 (1:750, NB100-142, Novus Biological), mouse anti-BRCA1 (1:500, sc-6954 D9, Santa Cruz Biotechnology), rabbit anti-53BP1 (1:500, ab172580, Abcam), goat anti-Mouse Alexa-594 (1:1000, A-21235, Thermo Fisher), goat anti-Rabbit Atto-647N (1:1000, 40839, Sigma).
Validation	- rabbit anti-MRE11 previously validated in HEK293T cells (https://www.science.org/doi/10.1126/science.aav9023).<br>- mouse anti-BRCA1 previously validated in U2OS and HeLa cells (https://datasheets.scbt.com/sc-6954.pdf)<br>- rabbit anti-53BP1 previously validated in 16 studies (https://www.abcam.com/53bp1-antibody-ab172580.html)<br>- goat anti-Mouse Alexa-594 widely validated in over 1200 studies (https://www.thermofisher.com/antibody/product/Goat-anti-Mouse-IgG-H-L-Cross-Adsorbed-Secondary-Antibody-Polyclonal/A-21235)<br>- goat anti-Rabbit Atto-647N validated in over 30 studies (https://www.sigmaaldrich.com/US/en/product/sigma/40839)

Eukaryotic cell lines

Policy information about cell lines

Cell line source(s)	HEK293T cells (ATCC® CRL-3216™) and K562 cells (ATCC® CCL-243™) are from ATCC. WTC-11 cells are from Coriell Institute (GM25256)
Authentication	None of the cell lines were formally authenticated by the authors. The appearance and growth characteristics matched expectations.
Mycoplasma contamination	Cell lines tested negative for mycoplasma contamination.

Commonly misidentified lines (See ICLAC register)	No commonly misidentified cells were used in this study.

Animals and other organisms

Policy information about studies involving animals; ARRIVE guidelines recommended for reporting animal research

Laboratory animals	8-10 week old male C57BL/6J mice.
Wild animals	No wild animals were used in this study.
Field-collected samples	No field-collected samples were used in this study.
Ethics oversight	All mouse studies were carried out in accordance with guidelines and approval of the Johns Hopkins University Animal Care and Use Committee (Protocol #MO20M274).

Note that full information on the approval of the study protocol must also be provided in the manuscript.

ChIP-seq

Data deposition

☒ Confirm that both raw and final processed data have been deposited in a public database such as GEO.

☒ Confirm that you have deposited or provided access to graph files (e.g. BED files) for the called peaks.

Data access links *May remain private before publication.*	Sequencing data: https://www.ncbi.nlm.nih.gov/bioproject/PRJNA801688/. Called peaks from BLENDER (https://github.com/staciawyman/blender) output accessible at: https://github.com/rogerzou/DSeqPlus/tree/main/peaks
Files in database submission	List of files displayed on website linked above.
Genome browser session (e.g. UCSC)	N/A

Methodology

Replicates	Ranging from 1-5 biological replicates.
Sequencing depth	10 million to 50 million paired-end reads.
Antibodies	anti-MRE11 (1:750, NB100-142, Novus Biological)
Peak calling parameters	bowtie2 -p 6 -q --local -X 1000
Data quality	Reads were filtered for mapping quality >= 25. Singleton reads, potential PCR duplicates and index reads were removed.
Software	bowtie2, macs2, samtools, custom code available at https://github.com/rogerzou/DseqPlus

Flow Cytometry

Plots

Confirm that:

☒ The axis labels state the marker and fluorochrome used (e.g. CD4-FITC).

☒ The axis scales are clearly visible. Include numbers along axes only for bottom left plot of group (a 'group' is an analysis of identical markers).

☒ All plots are contour plots with outliers or pseudocolor plots.

☒ A numerical value for number of cells or percentage (with statistics) is provided.

Methodology

Sample preparation	Surface staining for flow cytometry was performed by washing T cells in PBS, pH7.4, followed by staining with LIVE/DEAD™ Fixable Violet Stain (ThermoFisher) at recommended concentration for 30 minutes on ice in the dark. T cells were then resuspended in Cell Staining Buffer (BioLegend) plus relevant antibodies (APC anti-NGFR [BioLegend]) and PE-conjugated HLA-A*02:p53 R175H tetramer (NIH Tetramer Facility) for 30 minutes at room temperature in the dark. Cells were washed twice in Cell Staining Buffer before resuspension for analysis.
Instrument	Flow cytometric analysis was performed on an IntelliCyt iQue Screener PLUS VBR (Sartorius).

Software	FlowJo v.10 was used for flow cytometry data analysis.
Cell population abundance	A single cell suspension of activated primary human CD3+ T cells was edited by CRISPR/Cas HDR and expanded in the presence or absence of Ku-60648 or vehicle, then subjected to flow cytometric analysis to define the frequency of live single NGFR+/tetramer+ T cells. The abundance of this population was 8.4%-13.3% among CRISPR/Cas-edited and 0% among mock-edited purified primary human CD3+ T cells, respectively.
Gating strategy	The gating strategy used for primary human T cells is shown in Extended Data Figure 2. For all experiments, debris was first excluded by a morphology gate based on FSC-H and SSC-H. Non-singlets were excluded from analysis by a single cell gate based on FSC-H and FSC-A. Live cells were selected by gating on cells negative for staining with LIVE/DEAD Fixable Violet dye (405 nm excitation, Invitrogen). Anti-NGFR APC+/tetramer PE+ cells were gated to define successfully edited live, single T cells using appropriate compensation with single-stained controls.

☒ Tick this box to confirm that a figure exemplifying the gating strategy is provided in the Supplementary Information.

April 2020
